# Erratum to Circular RNAs and human glioma

**DOI:** 10.20892/j.issn.2095-3941.2019.0442

**Published:** 2020-02-15

**Authors:** Liu Jinglei, Kun Zhao, Nunu Huang, Nu Zhang

**Affiliations:** ^1^Department of Neurosurgery, The First Affiliated Hospital of Sun Yat-sen University, Guangdong Provincial Key Laboratory of Brain Function and Disease, Guangzhou 510080, China

In the published article^1^, we omitted the Acknowledgements section on page 21, and it should be as below. We apologize for the errors and for any confusion it may have caused.

## References

[1] Liu J, Zhao K, Huang N, Zhang N (2019). Circular RNAs and human glioma. Cancer Biol Med.

